# Changes in mastectomy rates at a Brazilian public hospital over 20 years (1989 to 2008)

**DOI:** 10.1590/S1516-31802012000600002

**Published:** 2013-01-18

**Authors:** Débora Balabram, Fábio Braga Araújo, Simone Souza Porto, Joyce Soares Rodrigues, Atila Silva Sousa, Arminda Lucia Siqueira, Helenice Gobbi

**Affiliations:** I MD, PhD Student. General Surgeon, Department of Anatomical Pathology, Faculdade de Medicina da Universidade Federal de Minas Gerais (UFMG), Belo Horizonte, Minas Gerais, Brazil.; II Medical Student, Department of Anatomical Pathology, Faculdade de Medicina da Universidade Federal de Minas Gerais (UFMG), Belo Horizonte, Minas Gerais, Brazil.; III Statistics Student, Department of Statistics, Instituto de Ciências Exatas, Universidade Federal de Minas Gerais (UFMG), Belo Horizonte, Minas Gerais, Brazil.; IV PhD. Associate Professor, Department of Statistics, Instituto de Ciências Exatas, Universidade Federal de Minas Gerais (UFMG), Belo Horizonte, Minas Gerais, Brazil.; V MD, PhD. Associate Professor, Department of Anatomical Pathology, Universidade Federal de Minas Gerais (UFMG), Belo Horizonte, Minas Gerais, Brazil.

**Keywords:** Mastectomy, Mastectomy, segmental, Breast neoplasms, Brazil, Neoplasm staging, Mastectomia, Mastectomia segmentar, Neoplasias da mama, Brasil, Estadiamento de neoplasias

## Abstract

**CONTEXT AND OBJECTIVE:**

Recently, breast-conserving surgery (BCS) has been replacing mastectomy for breast cancer treatment. The aim of this study was to evaluate the changes in mastectomy and BCS rates and the factors relating to these shifts.

**DESIGN AND SETTING:**

A retrospective study in a Brazilian public hospital.

**METHODS:**

Pathological records from female patients who underwent surgery for breast cancer at Hospital das Clínicas, Universidade Federal de Minas Gerais (HC-UFMG), between 1989 and 2008 were reviewed. The mastectomy and BCS rates were calculated. The chi-square test was used to assess factors associated with type of surgical treatment and to compare trends in treatment type over the years. Logistic regression was used for multivariate analysis.

**RESULTS:**

From 1989 to 2008, 2050 breast cancer surgical specimens were received in our service, corresponding to 1973 patients; 1324 (64.6%) of them were from mastectomy and 726 (35.4%) from BCS. A shift from mastectomy towards BCS was observed (P < 0.001). In multivariate analysis, earlier year of surgery (P < 0.001), larger tumor size (P < 0.001), having at least one positive axillary lymph node (P < 0.001) and patients’ age greater than 68 years (P = 0.007) were predictors of mastectomy.

**CONCLUSIONS:**

There was a shift from mastectomy towards BCS in our institution over the years. This may reflect consolidation of BCS (plus radiotherapy) as an equivalent treatment to mastectomy in terms of survival and a shift to earlier diagnosis for the disease.

## RESUMO

**CONTEXTO E OBJETIVO:**

Recentemente, a cirurgia conservadora tem substituído a mastectomia para tratamento cirúrgico do câncer de mama. O objetivo deste estudo é avaliar as mudanças nas taxas de mastectomia e cirurgia conservadora e os fatores relacionados a essas mudanças.

**TIPO DE ESTUDO E LOCAL:**

Estudo retrospectivo em hospital público brasileiro.

**MÉTODOS:**

Dados patológicos das pacientes submetidas a cirurgia para câncer de mama no Hospital das Clínicas, Universidade Federal de Minas Gerais (HC-UFMG), de 1989 a 2008, foram revistos. As taxas de mastectomia e cirurgia conservadora foram calculadas. O teste qui-quadrado foi usado para avaliar fatores relacionados ao tipo de tratamento cirúrgico empregado e comparar tendências de tipo de tratamento ao longo dos anos. Regressão logística foi usada para análise multivariada.

**RESULTADOS:**

De 1989 a 2008, 2.050 espécimes cirúrgicos de câncer de mama foram recebidos em nosso serviço, correspondentes a 1.973 pacientes; 1.324 (64,6%) correspondentes a mastectomia e 726 (35,4%) correspondentes a cirurgia conservadora. A cirurgia conservadora se tornou mais frequente ao longo dos anos (P < 0,001). Em análise multivariada, ano mais precoce da cirurgia (P < 0,001), maior tamanho tumoral (P < 0,001), ter ao menos um linfonodo axilar positivo (P < 0,001) e idade maior de 68 anos (P = 0,007) foram preditores de mastectomia.

**CONCLUSÕES:**

As cirurgias conservadoras se tornaram mais frequentes em relação às mastectomias ao longo dos anos. Isso pode refletir a consolidação da cirurgia conservadora (associada a radioterapia) como equivalente à mastectomia em termos de sobrevida, bem como diagnóstico mais precoce da doença.

## INTRODUCTION

Breast cancer is the most common cancer in women worldwide, and also the most common cause of cancer deaths in this group.^1^ It was estimated that Brazil would have 49,240 new cases of the disease in 2011.^2^ Mortality from breast cancer is still high in Brazil, mostly due to a delay in diagnosis.^1^^,^^2^^,^^3^^,^^4^

During the first half of the 20^th^ century, Halsted’s radical mastectomy and some variations of the procedure were used to treat the disease.^5^^,^^6^^,^^7^^,^^8^ In the 1970s, Fisher’s group in the United States and Veronesi’s group in Italy started comparing the prognoses of patients who had undergone breast-conserving surgery (BCS; wide local excision known as lumpectomy in the United States and quadrantectomy in Italy) and mastectomy for early-stage breast cancer. They showed that, when associated with radiotherapy, BCS equaled mastectomy regarding long-term survival.^9^^,^^10^^,^^11^^,^^12^^,^^13^^,^^14^ This led the United States National Institutes of Health to publish a consensus in 1991 stating that BCS could be used when possible.^15^ After this consensus had been published, mastectomy rates decreased to less than 50% in some countries.^16^^,^^17^^,^^18^^,^^19^^,^^20^^,^^21^^,^^22^

Recently, some American hospitals have reported an increase in mastectomy rates, although American and European statistics in general do not show this increase. ^16^^,^^17^^,^^18^^,^^19^^,^^20^^,^^21^^,^^22^^,^^23^^,^^24^^,^^25^ We have not found any studies in major databases (PubMed or SciELO) comparing the rates of different types of surgery for breast cancer in Brazilian public institutions.

Many factors are known to influence the decision regarding BCS versus mastectomy. Among them are the contraindications that are usually taken into consideration for BCS: multicentric tumors, inflammatory breast carcinoma, large tumor in relation to breast size, inability to obtain negative surgical margins, patient’s choice and contraindications for radiotherapy.^23^^,^^26^^,^^27^^,^^28^^,^^29^^,^^30^ Other factors are the surgeon’s preference, histopathological tumor type, positivity of axillary lymph nodes, healthcare availability, findings from imaging studies and genetic abnormalities.^17^^,^^19^^,^^31^^,^^32^ Thus, not all patients eligible for BCS receive breast conservation, and mastectomy rates vary greatly between cancer centers.^17^^,^^22^^,^^33^^,^^34^^,^^35^ The Clinical Hospital of the Federal University of Minas Gerais (Hospital das Clínicas, Universidade Federal de Minas Gerais, HC-UFMG), Belo Horizonte, Brazil, is a public general hospital and a reference center for breast cancer treatment.

## OBJECTIVE

The aim of this study was to evaluate the changes in mastectomy and BCS rates at HC-UFMG from 1989 to 2008, and the factors relating to these shifts.

## METHODS

A retrospective study comparing BCS and mastectomy rates at HC-UFMG from 1989 to 2008 was conducted. The study was approved by the UFMG Ethics Committee. The files from the Breast Pathology Laboratory of the UFMG Medical School were all reviewed. Specimens relating to surgical treatment of breast cancer were selected, and the following variables were recorded: pathological tumor size (pT), regional lymph node status (pN), gender, age, laterality (right or left), history of contralateral breast cancer, histopathological type (invasive ductal carcinoma not otherwise specified, IDC; invasive lobular carcinoma, ILC; ductal carcinoma *in situ*, DCIS; lobular carcinoma *in situ*, LCIS; and special type carcinomas), and type of surgery performed (mastectomy or BCS). The last procedure performed during the time period was selected (e.g. if the patient underwent breast conserving surgery and then needed a mastectomy because of margin involvement or tumor recurrence, only the mastectomy was recorded). Male patients (eight cases) and patients whose primary biopsy was performed in another institution and who did not have any additional tumor at the time of surgery (eighteen cases) were excluded. All other patients who underwent surgery at HC-UFMG were included in the study.

Tumor staging was performed in accordance with the American Joint Committee on Cancer (AJCC) Cancer Staging Manual of 2002.^36^

Statistical analysis was performed using the Statistical Package for the Social Sciences (SPSS) version 17.0 and Epi Info 2000 softwares. The chi-square test was used to compare frequencies of type of surgery versus laterality, age, pT, pN and histopathology. The chi-square test for a linear trend was used to compare the frequencies of types of surgery over the years of the study. Student’s t test was used to compare mean ages. The significance level was defined as 0.05.

Age cutoff points were defined by means of the CHAID (Chi-square Automatic Interaction Detector) algorithm using the type of surgery as the response variable.^37^

A logistic regression model with the type of surgery as the response variable was used to identify factors associated with mastectomy. Starting with the univariate analysis, variables with a statistical significance of less than 0.2 (P < 0.20) were included in the multiple logistic regression model. The stepwise backward algorithm was used to select the variables with a P value under 0.05.^38^ Estimates of odds ratios (OR) and the corresponding 95% confidence interval (95% CI) were obtained from univariate and multivariate analyses.^39^ Since some patients had bilateral tumors, the analysis was repeated after excluding these cases in order to confirm the adequacy of the model. The conclusions did not change through their removal, so these cases were kept. The goodness of fit of the regression model was assessed using the Hosmer and Lemeshow test.

## RESULTS

From 1989 to 2008, 1973 female patients underwent breast surgery, with records available in the Breast Pathology Laboratory of HC-UFMG. The patients’ ages ranged from 19 to 97 years (mean = 55.13 years; standard deviation, SD = 13.9 years). A total of 2050 breast cancer specimens were evaluated. One thousand three hundred and twenty-four (64.6%) of them were from mastectomies, and 726 (35.4%) were BCS specimens. Table 1 shows the distribution of frequencies according to the type of surgical procedure, history of contralateral breast cancer, laterality, pathological tumor size (pT), status of regional lymph nodes (pN), histopathology and age range.

**Table 1.** Characteristics of 2050 breast cancer specimens received from 1989 to 2008

Variable	Frequency
Type of surgery	
Breast-conserving surgery	726 (35.4%)
Mastectomy	1324 (64.6%)
Contralateral breast cancer	
No	1917 (93.5%)
Yes	133 (6.5%)
Laterality	
Right	948 (46.2%)
Left	1092 (53.3%)
Unknown	10 (0.5%)
pT	
*in situ*	182 (8.9%)
pT1	354 (17.3%)
pT2	680 (33.2%)
pT3	164 (8%)
pT4	243 (11.9%)
Unknown	427 (20.8%)
pN	
pN0	803 (39.2%)
pN1	437 (21.3%)
pN2	253 (12.3%)
pN3	220 (10.7%)
Unknown	337 (16.4%)
Pathology	
Invasive ductal carcinoma	1530 (74.6%)
Ductal carcinoma *in situ*	195 (9.5%)
Lobular carcinoma *in situ*	17 (0.8%)
Invasive lobular carcinoma	109 (5.3%)
Other	199 (9.7%)
Age	
? 68	1618 (78.9%)
> 68	397 (19.4%)
Unknown	35 (1.7%)
Total	2050 (100%)

**Figure 1.**
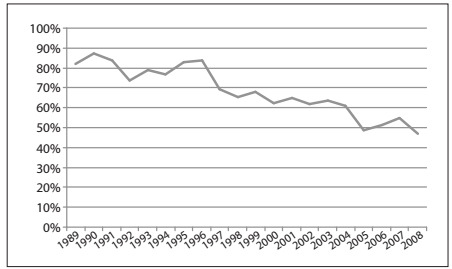
Percentage of mastectomies over time.

The total number of surgeries increased over the years, from 45 cases in 1989 to 160 in 2008 **(**Table 2**)**. The type of surgery performed shifted from a majority of radical mastectomy in 1989 (37 cases; 82.2%) to a minority in 2008 (75 cases; 46.9%; P < 0.001). Figure 1 shows the percentage of mastectomies over the years. In multivariate analysis, it was seen that the later the year was, the smaller the chances were that the patients would have undergone mastectomy (OR = 0.90 for each one year increase; CI = 0.88-0.93). Age did not differ between the two groups (mean age of patients undergoing BCS, 54.58 years; SD = 12.68 years; and undergoing mastectomy, 55.43 years; SD = 14.49 years). However, when age was categorized as 19 to 68 years and 69 to 97 years, as defined by CHAID analysis, women aged 69 to 97 years had a higher likelihood of undergoing mastectomy, in comparison with other age groups, both in univariate and in multivariate analyses (univariate: P < 0.001, OR = 1.64, CI = 1.28-2.11, Table 3; multivariate: P = 0.007, OR = 1.6, CI = 1.14-2.29, Table 4).

**Table 2.** Number (%) of specimens of breast-conserving surgery (BCS) and mastectomy operated between 1989 and 2008

Year	BCS	Mastectomy	Total
1989	8 (17.8)	37 (82.2)	45 (100)
1990	5 (12.8)	34 (87.2)	39 (100)
1991	7 (16.3)	36 (83.7)	43 (100)
1992	19 (26.4)	53 (73.6)	72 (100)
1993	17 (21.3)	63 (78.8)	80 (100)
1994	15 (23.1)	50 (76.9)	65 (100)
1995	13 (17.1)	63 (82.9)	76 (100)
1996	16 (16.3)	82 (83.7)	98 (100)
1997	31 (30.7)	70 (69.3)	101 (100)
1998	31 (34.8)	58 (65.2)	89 (100)
1999	35 (31.8)	75 (68.2)	110 (100)
2000	37 (37.8)	61 (62.2)	98 (100)
2001	42 (35.3)	77 (64.7)	119 (100)
2002	53 (38.1)	86 (61.9)	139 (100)
2003	50 (36.2)	88 (63.8)	138 (100)
2004	65 (38.9)	102 (61.1)	167 (100)
2005	50 (51.5)	47 (48.5)	97 (100)
2006	79 (48.5)	84 (51.5)	163 (100)
2007	68 (45)	83 (55)	151 (100)
2008	85 (53.1)	75 (46.9)	160 (100)
Total	726 (35.4)	1324 (64.6)	2050 (100)

**Table 3.** Univariate analyses on factors associated with breast-conserving surgery and mastectomy

		Breast-conserving surgery (%)	Mastectomy (%)	Total	P-value	Odds ratio (95% confidence interval)
History of contralateral breast cancer	No	674 (35.2)	1243 (64.8)	1917 (100)	0.358	1
	Yes	52 (39.1)	81 (60.9)	133 (100)		0.84 (0.58-1.23)
Total		726 (35.4)	1324 (64.6)	2050 (100)		
Laterality	Right	312 (32.9)	636 (67.1)	948 (100)	0.032	1
	Left	409 (37.5)	683 (62.5)	1092 (100)		0.82 (0.68-0.99)
Total		721 (35.3)	1319 (64.7)	2040 (100)		
pT	pTis	95 (52.2)	87 (47.8)	182 (100)	< 0.001	1.29 (0.89-1.88)
	pT1	207 (58.5)	147 (41.5)	354 (100)		1
	pT2	191 (28.1)	489 (71.9)	680 (100)		3.61 (2.73-4.76)
	pT3	18 (11)	146 (89)	164 (100)		11.42 (6.52-20.22)
	pT4	8 (3.3)	235 (96.7)	243 (100)		41.36 (19.12-93.27)
Total		519 (32)	1104 (68)	1623 (100)		
pN	pN0	323 (40.2)	480 (59.8)	803 (100)	< 0.001	1
	pN1	120 (27.5)	317 (72.5)	437 (100)		1.78 (1.37-2.31)
	pN2	37 (14.6)	216 (85.4)	253 (100)		3.93 (2.66-5.83)
	pN3	33 (15)	187 (85)	220 (100)		3.81 (2.52-5.78)
Total		513 (29.9)	1200 (70.1)	1713 (100)		
Histopathology	IDC	485 (31.7)	1045 (68.3)	1530 (100)	< 0.001	1
	DCIS	108 (55.4)	87 (44.6)	195 (100)		0.37 (0.27-0.51)
	Other	92 (46.2)	107 (53.8)	199 (100)		0.54 (0.4-0.74)
	ILC	29 (26.6)	80 (73.4)	109 (100)		1.28 (0.81-2.03)
	LCIS	12 (70.6)	5 (29.4)	17 (100)		0.19 (0.05-0.59)
Total		726 (35.4)	1324 (64.6)	2050 (100)		
Age	? 68	605 (37.4)	1013 (62.6)	1618 (100)	< 0.001	1
	> 68	106 (26.7)	291 (73.3)	397 (100)		1.64 (1.28-2.11)
Total		711 (35.3)	1304 (64.7)	2015 (100)		

IDC = invasive ductal carcinoma; DCIS = ductal carcinoma *in situ;* ILC = invasive lobular carcinoma; LCIS = lobular carcinoma *in situ.*

**Table 4.** Multivariate analysis on predictors of mastectomy versus breast-conserving surgery

Factor	P-value	Odds ratio
Year	< 0.001	0.90 (0.88-0.93)
pT1	< 0.001	1.00
pTis	< 0.001	4.41 (2.58-7.54)
pT2	< 0.001	3.16 (2.35-4.25)
pT3	< 0.001	10.45 (5.78-18.92)
pT4	< 0.001	38.66 (16.47-90.75)
Age > 68 years	0.007	1.62 (1.14-2.29)
Positive axillary lymph nodes	< 0.001	1.89 (1.44-2.47)

The most common type of tumor was IDC, with 1530 cases (74.6%). ILC accounted for 109 cases (5.3%), DCIS for 195 (9.5%), LCIS for 17 (0.8%) and special-type carcinomas for 199 cases (9.7%). DCIS, LCIS and special-type carcinomas (medullary, mucinous, papillary, secretory, metaplastic and apocrine carcinomas), were associated with a smaller mastectomy rate (OR for DCIS = 0.37, CI = 0.27-0.51; for LCIS = 0.19, CI = 0.05-0.59; and for special type carcinomas, OR = 0.54, CI = 0.4-0.74, Table 3) in univariate but not multivariate analyses.

A history of contralateral breast cancer (synchronous or metachronous), which occurred in 133 cases (6.5%), was not a predictor of mastectomy (P = 0.358) in univariate analysis (Tables 1 and 3).

Regarding tumor size, 182 cases (8.9%) were *in situ*, 354 cases (17.3%) measured less than two centimeters (T1), 681 cases (33.2%) measured from two to five centimeters (T2), 164 cases (8.0%) measured more than five centimeters (T3), 243 cases (11.9%) were infiltrating skin or chest wall (T4) and 427 cases (20.8%) could not be assessed in relation to tumor size, mostly due to previous incisional biopsies performed in earlier periods **(**Table 1**)**. Regional lymph nodes were negative for metastasis in 803 cases (39.2%); 437 cases (21.3%) were N1, 253 cases (12.3%) were N2 and 220 cases (10.7%) were N3 **(**Table 1**)**. In 337 cases (16.4%), lymph node status was unavailable, most likely because the procedures had been performed in other institutions. Tumor size was a predictor of mastectomy in univariate and multivariate analysis (P < 0.001 in both analyses; Tables 3 and 4). Having at least one positive axillary lymph node was also a predictor of mastectomy (P < 0.001 in both of them). The larger the tumor size was, the higher the odds were of undergoing mastectomy.

**Figure 2.**
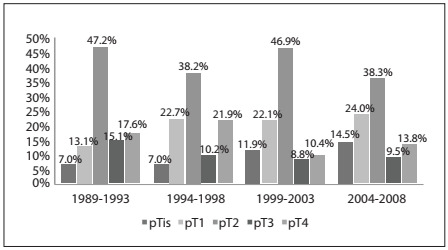
Proportions of pathological tumor sizes over time.

Pure forms of *in situ* tumors were more frequently diagnosed in later years. From 1989 to 1993, the proportion of patients with *in situ* tumors was 7% (14 cases), and from 2005 to 2008 that proportion rose to 14.5% (87 cases, P < 0.001; Figure 2). Also, the proportion of patients with a T1 tumor rose from 13.1% (26 cases) from 1989 to 1993, to 24% (144 cases) from 2004 to 2008. Figure 2 shows the proportions of pathological tumor sizes in four different time periods.

Regional lymph node status did not change significantly over the years. From 1989 to 1998, the percentage of cases with negative axillary lymph nodes was 46.9% (295 cases), versus 53.1% (334 cases) with at least one positive axillary lymph node; from 1999 to 2008, 508 (46.9%) cases had negative axillary nodes (P = 0.99), versus 576 cases (53.1%) with at least one positive lymph node.

In summary, the final regression model showed that use of mastectomy was associated with older age (> 68 years), earlier year of treatment, larger tumor size (pT) and positive regional lymph node status (pN). Histopathology was not significant in the model. The results from the Hosmer and Lemeshow test showed that the model fit was good (P = 0.251).

## DISCUSSION

Our study evaluated a series of women who were treated for breast cancer at a Brazilian public general hospital in the state of Minas Gerais that is also a reference center for breast cancer treatment. The mastectomy rates decreased significantly during the study period, from 82.2% in 1989 to 46.9% in 2008. The decrease in tumor size at diagnosis over the years, as well as the consolidation of BCS and the guidelines recommending its use when possible could be responsible for this decrease.^10^^,^^11^^,^^12^^,^^13^^,^^15^ In 2004, the Brazilian National Cancer Institute published a consensus stating that BCS could be used for tumors smaller than three centimeters.^40^ However, by the 2000s, developed countries had lower mastectomy rates, ranging from 35% to 46%.^16^^,^^17^^,^^18^

In a significant percentage of cases, we were unable to estimate the tumor size (20.8%) because multiple specimens were derived from the same tumor (incisional biopsies) or because some procedures had been performed in other institutions. We were also unable to estimate lymph node status in 16.4% of the patients, because they had not undergone surgical axillary staging or had undergone this procedure in another institution. Even though this could have affected our results, tumor size and lymph node status were strong independent predictors of the type of surgical treatment.

Other than tumor size and lymph node status, many other factors are known to affect the use of BCS or mastectomy.^17^^,^^18^^,^^20^^,^^23^^,^^31^^,^^32^^,^^33^^,^^34^^,^^35^^,^^41^ In our study, as in many other studies, older age was found to be a predictor of mastectomy. This differs from other studies, like those by Katipamula et al.^20^ (younger age was found to be a risk factor for mastectomy), McGuire et al.^23^ and Adkinsson et al.^25^ On the other hand, Reitsamer et al.^32^ showed that there was no correlation between age and type of surgery, and Zorzi et al.^18^ and Chagpar et al.^42^ showed higher mastectomy rates in older women. This may be explainable by different perception of breast importance in different cultural settings. It may also be attributable to our methodology, which selected only the last procedure performed on the patient. Therefore, the older woman may in fact have undergone BCS but might have had a recurrence later on and needed a mastectomy. Sixty-seven patients (5.1% of the mastectomies) first underwent breast conserving surgery and then mastectomy in a second intervention. Some authors have also reported that adjuvant therapy (such as chemotherapy) is less prescribed to older women, especially with a poor health status,^43^^,^^44^ and thus a more aggressive local therapy may have been chosen. It has been noticed that younger women have higher recurrence rates after BCS.^45^^,^^46^

Since the source of our study was pathological records, patients’ choices were not addressed although they could have influenced mastectomy rates.^22^^,^^28^^,^^35^^,^^47^

Some authors have concluded that, even though the public health system pays for mammograms, the adherence of women to screening mammograms is still low in Brazil, especially among less privileged women. Lima-Costa and Matos^3^ showed that, in 2003, only 43% of women between the ages of 50 and 69 years (the age recommended for screening mammograms according to the Brazilian National Cancer Institute^40^) had had a mammogram within the previous two years. Marchi and Gurgel^4^ showed that a higher percentage had had a first mammogram (68%), but that less than 50% of the patients were adherent to biannual screening. Moreover, women within the public system were less likely to comply with subsequent screening.^4^ This could be responsible for our high percentage of patients with locally advanced tumors (11.9% infiltrating the skin or chest wall). This differs from developed countries, such as the United States, Italy and the United Kingdom, in which most women undergo screening mammograms as recommended. ^16^^,^^17^^,^^18^

It may be possible that women with more advanced tumors are more frequently referred to our hospital, and therefore we have a higher than expected percentage of large tumors. On the other hand, even though still low, we are experiencing an increase in breast cancer screening, which has now become a focus of public health policies. SISMAMA (Information System for Breast Cancer Control), implemented by the Brazilian National Cancer Institute in 2009,^48^ is a tool for following up patients with abnormal breast examinations (imaging or pathology), and is also being used to direct funding for breast cancer screening.

In our study, IDC was associated with higher chances of mastectomy in univariate analyses than were DCIS, LCIS and special-type carcinomas. In multivariate analyses, the association was not significant. This differs from some studies, like those of Katipamula et al.,^20^ Chagpar et al.^42^ and Lee et al.,^30^ which showed higher mastectomy rates among patients with ILC.

Since the late 1980s,^15^ many developed countries have reported decreasing mastectomies rates for early breast cancer.^19^^,^^20^^,^^21^ However, no Brazilian data from either public or private hospitals concerning the type of surgery performed is available in the indexed literature. Through the present study, we have been able to conclude that in our hospital, which provides public care for breast cancer, this shift did not occur until recently. Moreover, we observed higher mastectomy rates for the same period than in developed countries such as the United States, Italy and the United Kingdom.^16^^,^^17^^,^^18^

Even though total mastectomy rates alone are not an accurate measurement for breast cancer care, they bring an insight into the complex issues involved in surgical management of the disease.^16^^,^^22^^,^^24^^,^^25^^,^^28^^,^^29^^,^^30^^,^^31^^,^^32^^,^^33^^,^^34^^,^^35^^,^^47^ In our study, one of these issues is the increasing frequency of early breast cancer at the time of surgery, which is a necessary condition for performing BCS, and is associated with better patient outcomes.^36^

## CONCLUSIONS

There was a clear shift from mastectomies towards BCS for breast cancer treatment at HC-UFMG over the 20 years of this study. This may reflect not only consolidation of BCS plus radiotherapy as an equivalent treatment to mastectomy in terms of overall survival, but also a shift to less locally advanced tumors at diagnosis, due to a higher percentage of women undergoing screening mammograms.
